# Editorial: Current knowledge and further development in the field of otoprotection

**DOI:** 10.3389/fneur.2023.1171439

**Published:** 2023-03-07

**Authors:** Joachim Schmutzhard, Anneliese Schrott-Fischer

**Affiliations:** Department of Otorhinolaryngology, Medical University of Innsbruck, Innsbruck, Austria

**Keywords:** otoprotection, smart watch, PEGylated IGF, nanoparticles, RNAs targeting NOX3

Over the next 30 years, up to 2.5 billion people will experience various degrees of hearing loss. Approximately 700 million people will be dependent on technical rehabilitation, like different hearing implant solutions. Thus, hearing impairment will become a medical and economic burden in the coming decades. Given this context, the emphasis on different otoprotective efforts is understandable and necessary. This special issue, “*Current knowledge and further development in the field of otoprotection*,” presents a selection of different papers approaching this important topic. A diverse range of approaches to tackling this topic is presented, from everyday usable solutions to clinical applications such as intraoperative monitoring or imaging to highly technical laboratory approaches. This spectrum depicts the wide approach taken by the community to this topic.

Nowadays, noise-induced hearing loss, resulting from increased daily noise exposure, is a major cause of acquired hearing loss. Awareness of individual excessive noise exposure is an important tool to protect the population from noise-induced hearing loss. As elaborated by Fischer et al., smart watches can play an important role in monitoring personal noise exposure and provide a widely available and cost-effective measure for otoprotection. Additionally, generating fundamental knowledge of the pathophysiological mechanisms underlying noise-induced hearing loss is a further milestone in understanding this unique burden. The development of therapeutic and protective options is aided by an improved understanding of noise-induced hearing loss. Cederholm et al. contributed by utilizing a type III intermediate filament peripherin gene knockout mouse model in a noise-induced hearing setup. The authors demonstrated a clear association between disruption of OHC-type II SGN sensory input, near elimination of MOC efferent-mediated contralateral suppression at moderate to high sound levels, and decreased otoprotection in noise-induced hearing loss.

Aside from such specific approaches, the field of otoprotection includes a variety of therapeutic approaches, ranging from growth factors to modulations of reactive oxygen species-generating enzymes. These otoprotective ideas show the breadth of creativity used in the development of various treatment modes. Reactive oxygen species, like NOX3, have an implicated role in acquired sensorineuronal hearing loss. Unfortunately, no specific pharmacological inhibitor is available so far. In a novel attempt, Nacher-Soler et al. elaborated on experimental molecular therapeutic access. A robust temporal NOX3 downregulation could be demonstrated by intracochlear delivery of NOX3-siRNAs. Various alternative therapeutic approaches have been evaluated so far. Growth factors have been shown to have a positive effect on the inner ear. This otoprotective branch has been further investigated by Bieniussa et al.. In a special progressive motor neuropathy mouse model, the deterioration of the auditory nerve and the outer hair cells could be diminished by the application of PEGylation insulin-like growth factor. Insulin-like growth factor PEGylation is a modification that prolongs the factor's half-life while maintaining full activity. Aside from studying the direct pharmacological effect of otoprotective agents, another area of focus was drug delivery to the inner ear. In the included review by Barbara et al., the large field of nanoparticles and their applicability in inner ear drug delivery was included. Nanoparticles have been proven to be safe, with no significant decrease in cell viability or signs of ototoxicity. This drug application form includes encapsulation, polymerization, surface functionalization, and enhanced drug release. Dexamethasone and antioxidants represent the most frequently used drugs in the evaluated studies.

In addition to drug identification and application strategies, the broad field of otoprotection extends to daily clinical routines, ranging from CI electrode insertion surveillance technologies to improved MRI technologies. Due to the expanding indication criteria for cochlea implantation candidacy, the procedure is now performed with significant residual hearing. Monitoring of residual hearing during the electrode insertion process is another otoprotective focus. Wimmer et al. evaluated electrode impedance variability as a potential biomarker for residual hearing. In 42 CI patients, the group associated a 1 kΩ increase with a 4.4 dB deterioration of residual hearing.

This special topic issue includes a variety of publications reflecting the breadth and diversity of otoprotective research.

## Author contributions

All authors listed have made a substantial, direct, and intellectual contribution to the work and approved it for publication.

